# Northern Ohio Dental Association

**Published:** 1864-06

**Authors:** 


### NORTHERN OHIO DENTAL ASSOCIATION.

The annual meeting of the Association was held in Cleveland, on Tuesday afternoon and evening of May 3d, 1864. President Dr. B. Strickland in the chair.

The following officers were elected for the coming year:

*President*—Dr. B. Strickland.

*Vice-President*—Dr. C. Palmer.

*Recording Secretary*—Dr. L. Buffett.

*Corresponding Secretary*—Dr. B. F. Robinson.

*Committee on Membership*—Drs. Palmer, Butler and Lyman.

Drs. Robinson, Buffett and Harrington were appointed delegates to the American Association. Drs. Terry, Siddell and Slosson as alternates.

Essays were read by Drs. Palmer, Lyman and Siddell.

Subject by Dr. Palmer—Filling Teeth; giving the manner of treating difficult cavities, and the claims of the different materials used in filling.

Subject of Dr. Lyman—Dental Associations.

Subject of Dr. Siddell—Cheap Dentistry.

A paper was read from Dr. Atkinson, on preparation, in which the Dr. clearly set forth the need of thorough preparation of all students; also the honest working of the profession.

The evening session was mostly spent in discussing the manner of destroying nerves, and the time and manner of filling fangs. After the discussion, Drs. Buffett, Butler, Slosson and Terry were appointed essayists for the next annual meeting.

On motion adjourned. The next annual meeting to be held in Warren, Ohio.

